# The impact of a standardised intramuscular sedation protocol for acute behavioural disturbance in the emergency department

**DOI:** 10.1186/1471-227X-10-14

**Published:** 2010-06-28

**Authors:** Leonie A Calver, Michael A Downes, Colin B Page, Jenni L Bryant, Geoffrey K Isbister

**Affiliations:** 1 Discipline of Clinical Pharmacology, University of Newcastle, New South Wales, Australia; 2 Department of Clinical Toxicology and Pharmacology, Calvary Mater Newcastle, New South Wales, Australia; 3 Emergency Department, Calvary Mater Newcastle, New South Wales, Australia; 4 Emergency Department, Princess Alexandra Hospital, Brisbane, Queensland, Australia; 5 Department of Liaison Psychiatry, Calvary Mater Newcastle, New South Wales, Australia

## Abstract

**Background:**

Acute behavioural disturbance (ABD) is an increasing problem in emergency departments. This study aimed to determine the impact of a structured intramuscular (IM) sedation protocol on the duration of ABD in the emergency department.

**Methods:**

A historical control study was undertaken comparing 58 patients who required physical restraint and parenteral sedation with the structured IM sedation protocol, to 73 historical controls treated predominantly by intravenous sedation, according to individual clinician preference. The primary outcome was the duration of the ABD defined as the time security staff were required. Secondary outcomes were the requirement for additional sedation, drug related-adverse effects and patient and staff injuries.

**Results:**

The median duration of the ABD in patients with the new sedation protocol was 21 minutes (IQR: 15 to 35 minutes; Range: 5 to 78 minutes) compared to a median duration of 30 minutes (IQR: 15 to 50 minutes; Range: 5 to 135 minutes) in the historical controls which was significantly different (p = 0.03). With IM sedation only 27 of 58 patients (47%; 95% CI: 34% to 60%) required further sedation compared to 64 of 73 historical controls (88%; 95%CI: 77% to 94%). There were six (10%) drug-related adverse events with the new IM protocol [oxygen desaturation (5), oxygen desaturation/airway obstruction (1)] compared to 10 (14%) in the historical controls [oxygen desaturation (5), hypoventilation (4) and aspiration (1)]. Injuries to staff occurred with three patients using the new sedation protocol and in seven of the historical controls. Two patients were injured during the new protocol and two of the historical controls.

**Conclusion:**

The use of a standardised IM sedation protocol was simple, more effective and as safe for management of ABD compared to predominantly intravenous sedation.

## Background

Acute behavioural disturbance (ABD) is a regular occurrence in emergency departments (ED) and is one of the commonest indications for sedation to be utilised in the ED[1]. There are numerous causes of ABD in the ED, but drug and alcohol intoxication or withdrawal, confusion and agitation related to behavioural disorders or threatening self harm or poisoning, are the most frequent[2,3]. The optimal goal in the management of patients with ABD is to ensure safety for the patient, staff and other patients.

Considerable literature focuses on the sedation of patients in psychiatric institutions[4-7] where most patients have psychotic illness, and the requirement for rapid sedation is less common. Despite the existence of numerous guidelines for sedation of aggressive patients in the ED[8,9], there are limited studies on this[3,10-15], predominantly focusing on comparing different drug types. There are few studies specifically examining structured approaches to sedating agitated patients[2] and no studies comparing different routes of administration of sedation in the ED. Currently numerous different sedative drugs and combinations of drugs are used, given variously by the intramuscular (IM) and the intravenous (IV) route. The lack of evidence often results in treatment choices being determined by individual staff preference resulting in little consistency in the management of these difficult patients.

As part of a clinical trial to compare different drugs for IM sedation in the ED, a structured approach to sedation was introduced which involved IM sedation only being used as the initial route of sedation. The same ED had previously used predominately IV sedation in this patient group[2]. This study aimed to investigate the impact of this structured approach for sedation on duration of ABD episodes, requirements for additional sedation and the effect on drug related adverse events.

## Methods

### Setting and study design

We undertook a historical control study to investigate the effect of the introduction of a standardised protocol for the sedation of violent behaviour in the ED that exclusively used the IM route of administration for sedation. Patients treated using the new IM sedation protocol were compared to historical controls. The historical controls were taken from the period prior when the existing practice was to predominantly use IV sedation. The structured IM sedation protocol was introduced as part of a clinical trial comparing droperidol (10 mg), midazolam (10 mg) and a combination of droperidol (5 mg) and midazolam (5 mg). The clinical trial is described in detail elsewhere. Ethics approval was obtained for the historical control study from the Human Research Ethics Committee.

The hospital where the study was undertaken has a tertiary toxicology unit, and although there are only 27,000 presentation to the ED annually, there is a high proportion of patients with agitation, delirium, aggression and acute behavioural disorders because the hospital provides a regional clinical toxicology service and Drug and Alcohol Unit[2].

### Selection of Participants

The study compared patients treated with the new structured IM sedation protocol during an eight month period from August 2008 to March 2009 to a group of historical control patients sedated in the ED in the eight month period immediately before the protocol was introduced (November 2007 to June 2008). The structured IM sedation protocol consisted of:

1.An intramuscular injection of the clinical trial drug, which was labelled and kept in the ED.

2.A defined approach to monitoring of the patient's vital signs over a six hour period

3.The introduction and use of a sedation score to be included as part of the standard observation of the patient

4.Recording of further sedation, adverse events, staff or patient injury for all patients.

5.Route and type of additional sedation was dictated by the treating clinician.

**Figure 1:**
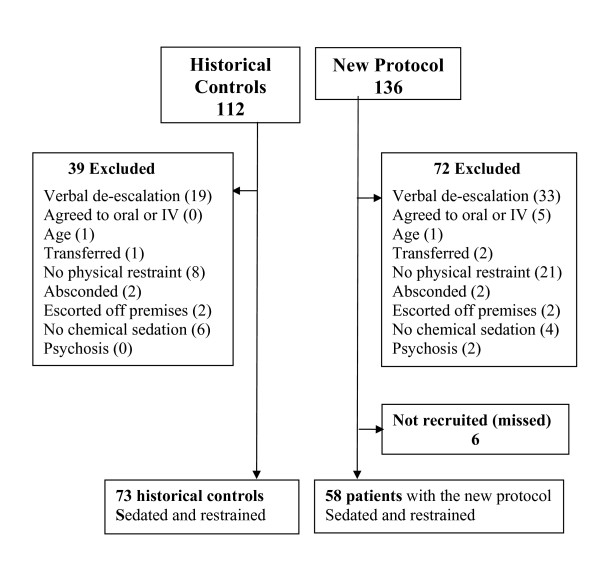
**Flow chart illustrating all patients with a ABD recorded in the security log and which were during the new protocol or included in the historical control group**.

Inclusion criteria for both the historical controls (use of predominantly IV parenteral sedation) and the intervention group (IM sedation only) were that the patient required both physical and chemical restraint, the patient did not consent to IV or oral sedation and they required the presence of the hospital security. To identify and ensure that the historical control group was similar to patients during the new IM sedation protocol we accessed the hospital security log for both time periods. The security log documents all security responses to ABD in the ED and has previously been shown to be the most accurate record of patients with ABD[2]. Medical records were retrieved for all patients who had required security to attend the ED and only patients meeting the inclusion criteria were included. Exclusion criteria were successful verbal de-escalation, agreement to oral or IV sedation, previous administration of other sedative medication or the patient did not remain in the ED (escorted off premises by police, absconded) (Figure 1).

### Data Collection and Processing

Data was recorded prospectively as part of the new structured sedation protocol on standardised datasheets which were then entered into a relational database. The duration of the ABD was taken from the security log. All outcomes were defined prior to the introduction of the sedation protocol. Identical data items were extracted retrospectively from the medical records of patients in the historical control group. The extraction process was undertaken by one investigator (LAC) but was reviewed by a second investigator (GKI) for the first ten patients. There were no differences in the recording of the outcomes between the two investigators.

The following data were included for the study analysis: patient demographic characteristics (age, sex), cause of ABD, duration of the ABD episode, any use of additional sedation in the patient including the time of administration, drug related adverse effects and injuries to patients and staff.

### Methods of Measurement

Information was recorded by an investigator or research nurse for a six hour period after initial sedation. For historical controls the medical record was used to obtain information from the standard ED observation chart. For all patients including historical controls, the duration of the ABD was extracted from the security log based on the time from the initial call time to security to the "all clear" time when they are released from attendance. The security staff defined an "all clear" when the patient is safely secured by all four limbs, a mask is in situ if the patient is spitting, the patient is sedated or settling and the verbal abuse is abating or ceased. This is determined in consultation with the clinical staff present at the time. The security staff and ED clinical staff were not aware that the duration of ABD was the primary outcome for the study. During the new sedation protocol, additional medications used and adverse events were recorded prospectively and checked with the patient's medication chart and medical record. For historical controls this information was extracted from the medication chart and medical record. The data for the historical controls and patients in the intervention period were the same because it is mandatory routine patient documentation.

### Main Interventions

The intervention was the introduction of a structured IM sedation protocol for ABD patients in the ED that involved initial sedation via the IM route with pre-determined medications [droperidol (10 mg), midazolam (10 mg) or a combination of droperidol (5 mg) and midazolam (5 mg)]. Prior to the study the use of sedation, including the drug type used and the route of administration was dictated by either the treating ED doctor or the consultant emergency physician or clinical toxicologist responsible for the patient. However, the vast majority of sedation used was administered via the IV route, rather than the IM route, and a selection of either benzodiazepines, antipsychotics or a combination of both were used in varying doses in each patient[2]. Recruitment to the prospective study was assisted by in-servicing which helped to ensured that all of the patients who required parenteral sedation and mechanical restraint were recruited to the study due to staff awareness and only six were missed (Figure 1).

### Main Outcomes

The primary outcome was the duration in minutes of the ABD comparing patients during the new intervention to the historical controls. Other outcomes included the requirement for additional sedation and adverse effects from the sedative medication, which includes both patient and staff incidents. Additional sedation included further sedation required to obtain initial sedation as well as re-sedation if the patient re-emerged still agitated. Re-sedation was defined as sedation after an interval exceeding one hour where no further sedation was given during that time, based on repeat sedation in a previous study[2]. Sedative related adverse effects included any episodes of oxygen desaturation (< 90%), hypoventilation (respiratory rate < 12), requirement for airway intervention or support, arrhythmias or hypotension. Staff or patient injuries were also determined for both groups and were reported during both periods as per hospital guidelines for incident monitoring.

### Data Analysis

Medians and interquartile ranges (IQR) are reported for all continuous variables. Percentages are reported for dichotomous outcomes with 95% confidence intervals (CI). Comparison of continuous variables between the two groups was performed using the non-parametric Mann-Whitney test. Statistical analysis was performed using Prism 5.01 (GraphPad Software Inc).

## Results

There were 58 patients recruited during 8 month period where the new IM sedation protocol was used. These were compared to 73 historical control patients that similarly required parenteral sedation in the 8 month period prior to the new IM protocol. Only 20 of the 73 historical controls (27%) initially received IM sedation, compared to all patients for the IM sedation protocol. Baseline characteristics for both groups are compared in Table 1 and are similar except for the larger proportion of males during the new protocol.

**Table 1:** Baseline characteristics of the historical control patients compared to patients with the new protocol of intramuscular sedation

	Historical Controls (N = 73)	New Protocol (N = 58)
**Age (median, IQR)**	30 years (23 to 38 years)	32 years (26 to 43 years)
**Sex (male)**	35 (48%)	32 (60%)
Initial intramuscular sedation*	20 (27%)	58 (100%)
**Benzodiazepines**	63 (86%)	67%^†^

IQR - interquartile range; ABD - acute behavioural disturbance; *The remainder received IV sedation (53). ^†^Exact number unknown but based on randomisation approximately one third received 10mg midazolam and one third 5mg midazolam and one third only droperidol.

**Figure 2:**
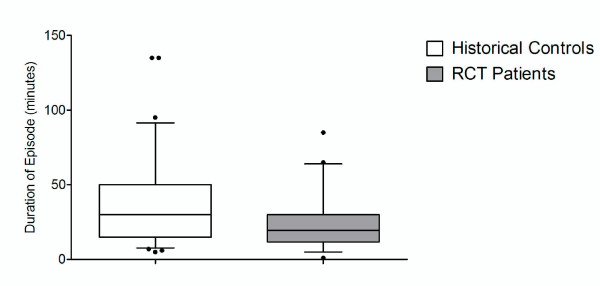
**Box and whisker plots showing the duration of the ABD comparing historical control patients to patients with the new sedation protocol**. The whiskers are the 5^th ^and 95^th ^percentiles, the box the interquartile range, extreme outliers are filled circles and the median by the full line across the box.

The median duration of the ABD in patients with the new sedation protocol was 21 minutes (IQR: 15 to 35 minutes; Range: 5 to 78 minutes) compared to a median duration of 30 minutes (IQR: 15 to 50 minutes; Range: 5 to 135 minutes) in the historical controls which was significantly different (p = 0.03) [Figure 2].

With the new IM sedation protocol 27 of the 58 patients (47%; 95%CI: 34% to 60%) required further sedative medication at any time compared to 64 of the 73 historical control patients (88%; 95%CI: 77% to 94%). The increased number of historical controls requiring further sedation was both for failed sedation in the initial period; and for re-sedation, as follows: 14 of 58 patients (24%; 95% CI: 14% to 37%) required further additional sedation compared to 47 of 73 historical controls (64%; 95% CI: 52% to 75%). The number of patients that required re-sedation with the new IM sedation protocol was 18 of 58 patients (31%; 95% CI: 20% to 45%) compared to 36 of 73 historical control patients (49%; 95% CI: 38% to 61%).

**Figure 3:**
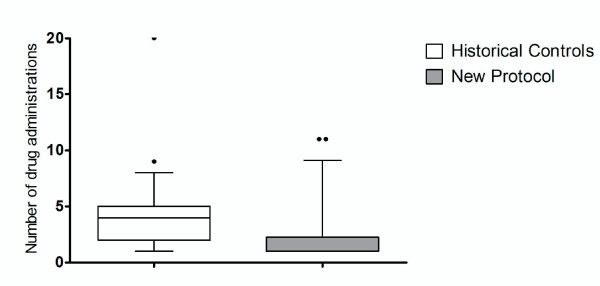
**Box and whiskers plot showing the number of total drug administration, including the initial sedation, comparing historical control patients to patients with the new sedation protocol**. The whiskers are the 5^th ^and 95^th ^percentiles, the box the interquartile range, extreme outliers are filled circles and the median by the full line across the box.

Of the 36 historical control patients re-sedated, 27 were re-sedated once, five re-sedated twice, two re-sedated three times and two re-sedated four times. In comparison, of the 18 patients with the new IM sedation protocol re-sedated, eleven were re-sedated once, three re-sedated twice, three re-sedated three times and one re-sedated six times. Figure 3 provides the total number of sedative drug administrations for both groups of patients. There were six (10%; 95% CI: 4% to 21%) sedative drug-related adverse events with the new IM protocol [oxygen desaturation (4), oxygen desaturation/airway obstruction (1), oxygen desaturation and atrial fibrillation (1)] compared to 10 (14%; 95% CI: 7% to 24%) in the historical controls [oxygen desaturation (5), hypoventilation (4) and aspiration (1)]. Injuries to staff occurred with three patients using the new sedation protocol and in seven cases with the historical controls. There were two patients injured during the new IM sedation protocol and two of the historical controls.

## Discussion

The study shows that a structured approach to sedation of ABD by using the IM route resulted in a reduced duration of ABD and less additional medication for sedation in the initial and subsequent episodes, compared to existing practice with predominantly IV sedation. In addition this was achieved without an increase in adverse events. This approach using the IM route has clear advantages because it means that sedation can be initiated rapidly in these dangerous patients who require mechanical restraint without gaining IV access. This will potentially reduce the risk of injury to staff and patients.

The reduced duration of the ABD, regardless of which drug was administered, is predominantly due to the fact that a structured IM protocol meant that the dose and route were established and treatment could be initiated immediately, often at the nursing staff's suggestion. In addition, IM sedation can be given rapidly without the need to gain IV access. The reduced time may therefore be a product of the practicalities of rapidly administering sedative medication - no need for IV access, rapid decision making and mechanical restraint - rather than the pharmacokinetics of the route of administration.

There is the need for a clear distinction between the duration of the ABD as opposed to the time to sedation. The ABD duration is measured from when the security staff are called to the ED. This can often include the time from when the patient is still ambulant or when the patient arrives in the ambulance or with police escort, until the "all clear" when security is no longer required. The time to sedation is the time it takes to achieve effective sedation from the time of drug administration and is usually measured using some form of sedation score. The fact that the IM route reduces the duration of the ABD is more likely due to shortening the time until administration rather than the time from administration to sedation. In this study the time to sedation could not be determined for the historical control group because sedation scores were only introduced as part of the new protocol.

Similar numbers of sedative related adverse effects with the new IM sedation protocol compared to historical controls was important because one of the concerns about using IM sedation is the risk of over-sedation. One study comparing IM ziprasidone, droperidol and midazolam reported respiratory depression in 15% of patients[3], similar to our study. Another study of high dose midazolam in acutely agitated ED patients, where two thirds received it via the IM route, reported adverse events in eight of 61 patients (13%)[16]. The drug was not known for each patient in our study and may differ between those receiving droperidol, midazolam or both.

There were limitations to the study because of the use of historical controls. Every attempt was made to ensure the two groups were similar (Figure 2, Table 2). However, the fact that sedative medications were not given in a standard way in the historical control group meant that the proportion who received benzodiazepines, antipsychotics and medication by the intramuscular route differed between the controls and the intervention group (Table 1). Potentially the success of the standardised intramuscular sedation protocol may have been because more patients received droperidol. This may explain the reduced requirement for additional sedation in the intervention group[3,14]. However, it is unlikely that more patients receiving droperidol in the intervention group accounts for the shortened duration of ABD because droperidol does not have a more rapid onset of action than benzodiazepines[10].

**Table 2:** Reasons for acute behavioural disturbance (ABD) in the historical controls and patients with the new protocol

Reason for ABD	Historical Controls	New Protocol
Alcohol intoxication	31 (42%)	14 (24%)
Deliberate self-harm	21 (28%)	20 (34%)
Deliberate self-harm and alcohol intoxication	9 (12%)	12 (21%)
Drug induced delirium/agitation*	9 (12%)	9 (15%)
Psychosis	0	2 (3%)
Personality disorder	0	1 (2%)
Drug withdrawal	0	0
Other (medical)	3 (4%)^†^	0

* including amphetamines, olanzapine; ^† ^urinary tract infection, head injury and hepatic encephalopathy

There were differences in the source of the data between the historical controls and the interventional group. However, the main outcomes in the study were determined prior to the new protocol being introduced and the retrospective data extraction for the historical controls. The duration of ABD was taken from the security log for both the historical controls and patients during the intervention period. The security staff were unaware that these times were used as the primary outcome for the study and there is no reason believe that the times were recorded differently in each period. The reliability of the recording of drug related adverse effects in the historical controls was dependent upon the accuracy of documentation by the clinical staff in the medical record. Therefore, there is a likelihood that some patients with or without adverse effects were missed in the historical controls. This would only underestimate the adverse effects in the historical controls because adverse events were prospectively monitored with the new sedation protocol and recorded on the data sheets. Information recorded regarding additional sedation is likely to be accurate for the historical controls because sedative medication is unlikely to be given without a written order.

There is a reasonable possibility that the reduction in ABD time and decreased need for additional sedation was in part due to research being undertaken with a study nurse being available to assist with data collection 24 hours a day - the Hawthorne effect. Even so, this is not necessarily a limitation and demonstrates that a structured approach to ABD with additional staffing provides improved sedation and treatment of these patients. However, the on-call staff members took approximately 20 to 30 minutes to arrive in the ED to assist with the data collection and in most cases the ABD was controlled and the security all clear called prior to their arrival.

Titrated intravenous sedation may have in fact been the intention in some historical control patients and therefore not considered a negative outcome. However, the time taken to give further sedation requires additional clinical time as it necessitates the presence of a medical officer and further ongoing 5 minutely observations by nursing staff. The patient's distress and struggle also continue to be prolonged in the case of repeated sedation attempts which are not in the patient's best interest. This delay in achieving sedation exposes the already chaotic ED to further disruption and increases risk of staff injury.

It is difficult to determine if the difference in the duration of ABD of 9 minutes is clinically significant and no previous studies have defined this. However, many would consider even 5 minutes in which a patient remains violent and aggressive and requiring security staff as being important. More importantly, the study shows that an IM sedation protocol is not inferior to a previously predominantly IV sedation and that such an approach is a feasible and safe alternative.

This study has potential relevance to regional hospitals which do not have the luxury of multiple security guards or even sufficient staff to adequately contain many of these patients. In this setting, an IM sedation protocol that does not require initial IV access, that acts reasonably rapidly and is safe, would be highly beneficial. With appropriate studies, such an approach may also be extended to other settings, such as psychiatric hospitals or the pre-hospital setting[11,13].

However, it may not be possible to immediately generalise these results to some other settings, based on the population of patients studied. The cause of ABD will differ with some hospitals having larger numbers of recreational overdoses, including amphetamine toxicity[14], compared to the predominant population of patients with deliberate self-harm and alcohol intoxication in our study[2]. It will be important to confirm this work to include other groups of patients because there is no reason that IM sedation should not be just as effective based on the fact that it appears to be the practicalities of administration rather than the drug type or administration route pharmacokinetics.

Further investigation is required to determine the best approach to the patient who fails initial sedation and/or requires several re-sedation episodes. The major problem with these patients is whether it is appropriate to repeat IM sedation, and if this will result in over-sedation. Because these patients remain violent and dangerous and it may still be difficult to obtain IV access, further IM sedation would be preferable if it can be shown to be safe and effective.

## Conclusion

We have shown that a structured approach to sedating agitated patients in the ED, where all initial doses of sedation were given IM, was simple, more effective and as safe for the management of ABD compared to the prior practice of using predominantly IV sedation. The duration of the ABD was shortened, less medication was used for additional sedation, and there were no increase in sedative related adverse events. The benefits of using the IM route in the ED may be translated to other settings with patients with ABD given the advantage of increased safety and effectiveness.

## Competing interests

The authors declare that they have no competing interests.

## Authors' contributions

LAC and GKI designed the study in consultation with MAD, CBP; LAC undertook data extraction; GKI, LAC and CBP undertook the analysis; LAC wrote the paper with GKI and MAD, CBP and JLB reviewed drafts. All authors read and approved the final manuscript. GKI takes responsibility for the study.

## Pre-publication history

The pre-publication history for this paper can be accessed here:

http://www.biomedcentral.com/1471-227X/10/14/prepub
